# Biomonitoring of parabens in wild boars through hair samples analysis

**DOI:** 10.1371/journal.pone.0297938

**Published:** 2024-02-21

**Authors:** Sławomir Gonkowski, Manolis Tzatzarakis, Elena Vakonaki, Elena Meschini, László Könyves, Liliana Rytel

**Affiliations:** 1 Department of Clinical Physiology, Faculty of Veterinary Medicine, University of Warmia and Mazury in Olsztyn, Olsztyn, Poland; 2 Laboratory of Toxicology, School of Medicine, University of Crete, Heraklion, Crete, Greece; 3 Department of Animal Hygiene, Herd Health and Mobile Clinic, University of Veterinary Medicine, Budapest, Hungary; 4 Department and Clinic of Internal Diseases, Faculty of Veterinary Medicine, University of Warmia and Mazury in Olsztyn, Olsztyn, Poland; Universidade Federal de Minas Gerais, BRAZIL

## Abstract

Parabens are compounds widely utilized in the industry as preservative additives to personal care products, cosmetics and food. They pollute the environment and penetrate to the living organisms through the digestive tract, respiratory system and skin. Till now the knowledge about exposure of terrestrial wild mammals to parabens is extremely scarce. Therefore, this study for the first time assessed the concentration levels of five parabens commonly used in industry (methylparaben—MeP, ethylparaben—EtP propylparaben–PrP, benzylparaben -BeP and butylparaben–BuP). Substances have been analyzed in hair samples collected from wild boars using liquid chromatography-mass spectrometry (LC-MS) method. The hair is a matrix, which allows to study long-term exposure of organisms to parabens. During this study MeP was noted in 96.3% of samples with mean 88.3±72.9 pg/mg, PrP in 87.0% of samples with mean 8.5±3.3 pg/mg, BeP in 44.4% of samples with mean 17.2±12.3 pg/mg and EtP in 11.1% of samples with mean 17.2±4.8 pg/mg. In turn BuP was noted only in 3.7% of samples with concentration levels below limit of quantification (2.6 pg/mg). Statistically significant intragender differences in parabens levels have not been noted. Only BeP concentration levels depended on industrialization and density of human population of area, where the animals lived. This study indicates that wild boars are exposed to parabens, especially to MeP and PrP, and analysis of the hair seems to be a useful tool of biomonitoring of parabens in wild mammals.

## 1. Introduction

Parabens are a large group of substances, that are chemically esters of parahydroxybenzoic acid (PHBA), differing from each other by functional groups, which may take the form of an alkyl chain or an aromatic ring [1]. Parabens have preservative properties, including fungistatic, anti-yest and anti-mold effects [2]. Moreover, in the lesser extent they also show an antibacterial activity [3]. Due to these properties, since the first half of the 20^th^ century parabens are globally used in various branches of industry, as preservative additives to personal care products, cosmetics, food and medicines [1, 2, 4]. Among parabens, the most commonly used are methyl paraben (MeP), ethyl paraben (EtP), propyl paraben (PrP), butyl paraben (BuP) and benzyl paraben (BeP) which are found, among others, in the shampoo, hair fixing gels, lipsticks, food packaging, baby wipes and various type of the food such as cereal-based snacks, dried meats, beer, sauces, desserts, soft drinks and jams [1, 2, 5, 6].

Parabens are also common in the environment. Some of them are of natural origin—produced by certain types of plants and bacteria, but the vast majority of parabens in the environment are the result of anthropogenic activity and using of these compounds in the industry [2]. Till now, the presence of parabens has been noted in the soil, surface water, air and dust in various parts of the world [7–9]. Moreover, these substances can penetrate to the living organisms through the digestive tract, lungs and skin [1, 2].

The influence of parabens on the living organisms is still a matter of discussion [2]. Until recently parabens were considered to be completely safe compounds without any negative impact for living organisms. However, the recent studies have found that parabens may negatively affect the endocrine system, and therefore they are classified as endocrine disruptors [1, 2]. Moreover, parabens show cytotoxic and genotoxic activities and may negatively affect various internal systems, including among others reproductive, immune and nervous systems [10–12]. In addition, some observations have confirmed connections between exposure to parabens and obesity, allergies, inflammatory processes, developmental diseases and cancer [5, 13–15].

Due to mentioned above adverse effects of parabens, the monitoring of exposure to these compounds is becoming an increasingly important issue in modern toxicology. Till now, the majority of studies concern exposure to parabens in humans, in which these compounds have been identified in various matrices, including among others the blood serum, urine, breast milk, hair, placenta and seminal plasma [16–18]. It has also been found that the degree of human exposure to parabens clearly depend on the region, where studies have been performed [8]. Parabens have also been described in domestic animals, namely in the urine of dogs, cats and cows, bovine row milk and canine fur [19–22].

Contrary to humans and domestic animals, the knowledge on the wild animals exposure to parabens is relatively scanty and limited mainly to the aquatic animals [23–26]. Only single studies concern the biomonitoring of parabens in wild terrestrial mammals. According to the best knowledge of the authors there are only three such studies describes parabens in the liver of the polar bear [27], liver and kidney of the black bear [28] and bat guano [29]. Moreover, two mentioned above studies on the bears have been conducted on a very limited number of samples (not exceeding ten) [27, 28]. On the other hand, due to the fact that pollution of the environment with parabens is widespread [8], these substances with high probability can negatively affect not only water organisms, but also the health status and hence population size of terrestrial animals. Therefore, monitoring of parabens in populations of wild terrestrial mammals seems to be very important in modern environmental toxicology.

It should be pointed out that in addition to the classic matrices, such as blood serum and urine [30, 31], other matrices (like hair, nails or seminal plasma) are more and more often used to determine parabens [16, 17]. The hair samples analysis is relatively new method to evaluate the degree of exposure to parabens, but it seems to be of special significance [32–34]. Previous studies on parabens and other endocrine disruptors polluting the environment have found that results obtained during hair analysis are similar in terms of sensitivity and repeatability to results obtained in studies on blood serum and/or urine samples [33, 35, 36] Simultaneously hair samples are very easy to collect, long term storage and transport even over long distances. The particular advantage of hair samples analysis is the fact that parabens and other pollutants accumulate in the hair for long time, and therefore such analysis is better to study on long-term environmental exposure than analysis of blood or urine, in which levels of pollutants are often subject to large short-term fluctuations [34]. Moreover substances levels in the hair do not change dramatically after the death of the organism, and therefore samples may also be collected from dead animals. However, according to the best knowledge of the authors, till now hair samples have been used to biomonitoring of parabens only it humans [32–34] and dogs [22], and despite promising results obtained in these investigations, this matrix has never been used to study on parabens in wild animals. On the other hand, hair analysis may be important in studies on the exposure of wild animals to parabens and other endocrine disruptors, because it is the ability to determine of these substances levels in the samples collected even from dead animals found in the natural environment.

It should be emphasized that the selection of parabens and wild boar for this research was not accidental. Among a wide range of endocrine disrupting chemicals polluting the environment occupy a special place. On one side, as mentioned above, they are used on massive scale in the cosmetic, plastics, food, pharmaceuticals and other industries [1, 2, 4] and therefore pollute the natural environment to a significant extent [7–9]. On the other hand, until recently parabens were considered to be non-toxic, and their multidirectional adverse effects have been described relatively recently [1, 2]. For this reason the knowledge about wild animal exposure to parabens is much more limited than knowledge about other endocrine active substances, which toxicity is better known.

In turn, wild boars are a specific mammal species. Their population is relatively large, and many individuals more and more often live and feed in the immediate vicinity of cities [37, 38]. For this reason, wild boars are often exposed to anthropogenic toxic substances polluting the environment, and therefore analysis of samples from this animal species may be a good way to monitor the degree of environmental pollution. Moreover, it is known that parabens as endocrine disruptors may affect the immune system [1, 39], and immune disorders may result in an increased incidence of bacterial and viral diseases. In this case, wild boars living in cities, which often show the presence of pathogenic microorganisms [40], may become an even greater threat to people and domestic animals.

Given the above, the aim of the present study was to determine for the first time the exposure of wild boars living in various regions of Poland (which differ in the respect of industrialization and human population density) to selected most commonly parabens, such as MeP, EtP, PrP, BuP and BeP through the analysis of the hair samples. The study on the one side allows to determine the exposure of wild boars to selected parabens and on other side shows usefulness of the hair samples to achieve this goal.

## 2. Materials and methods

### 2.1. Reagents

The following reagents were used during the present research: 1) MePB, EtPB, PrBP, BuPB, BePB (all ≥ 99%), and ammonium acetate (≥ 98%) from Sigma-Aldrich (St. Louis, MO, USA); 2) methanol and acetonitrile (LC-MS grade) from Fischer Chemicals, Loughborough, UK; 3) phenobarbital (IS) from Lipomed AG, Arlesheim, Switzerland; 5) ultrapure water produced by Merck’s Direct-Q 3UVwater purification system (Darmstadt, Germany).

### 2.2. Sample collection

**Fig 1:**
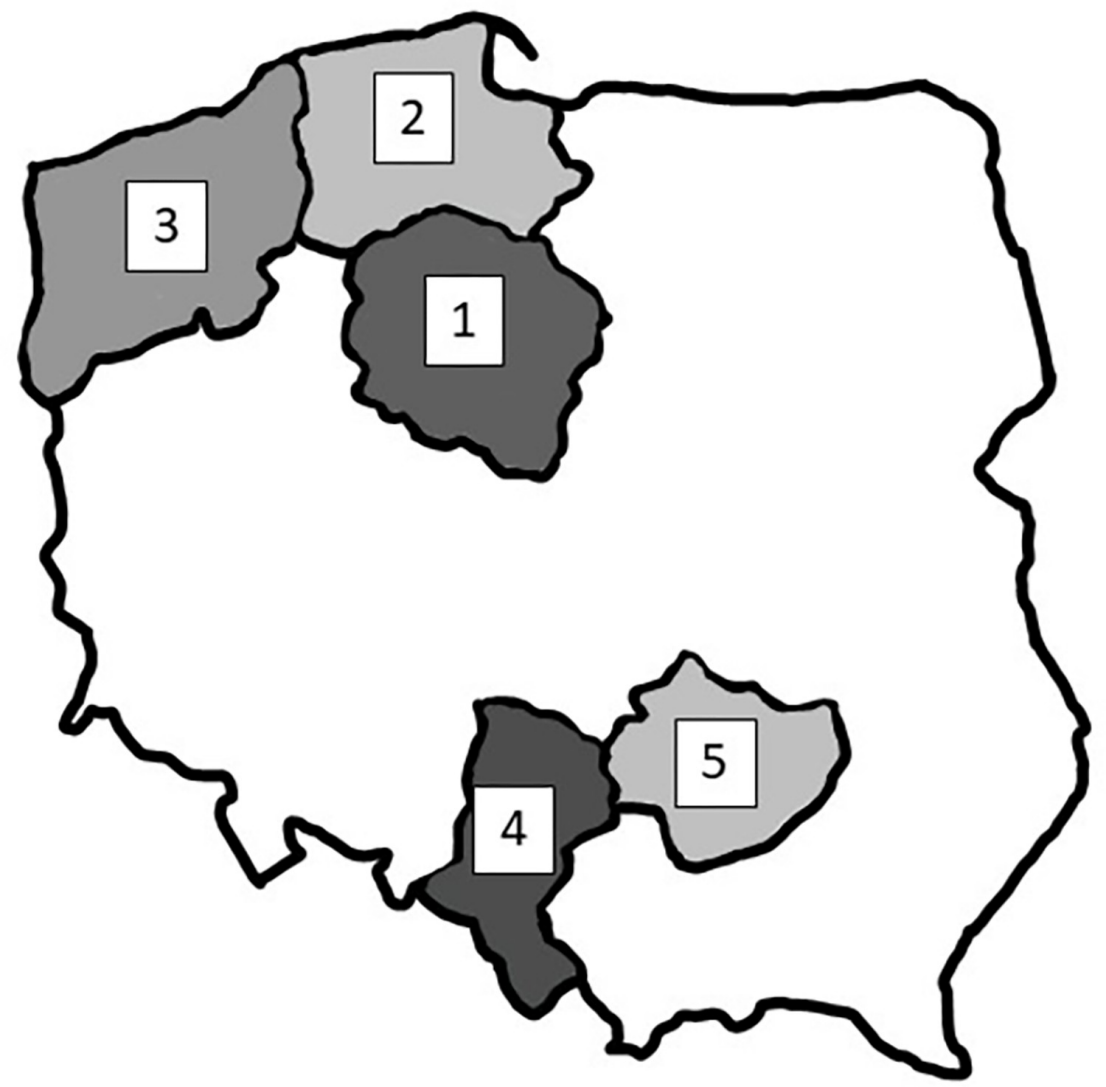
Hair samples collection sites.

Hair samples were collected from 54 adult wild boars (29 males and 25 females) hunted during legal hunting organized by Polish Hunting Association in years 2020–2022. The hunting took place in various regions of Poland, such as Kuyavian-Pomeranian, West Pomeranian, Pomeranian, Silesian and Holy Cross Voivodeships that differ in terms of the industrialization and human population density (Fig 1, Table 1). A voivodeship is the highest-level administrative division of Poland, corresponding to a province in many other countries, and Poland is divided into 16 voivodeships.

**Table 1:** Voivodeships included into the study.

No.	Name of voivodeship	Degree of industrialiaztion ^1)^	Human population density (number of persons/km^2^)^2)^
1	Kuyavian-Pomeranian	Medium industrialized	114
2	Pomeranian	Highly industrialized	128
3	West Pomeranian	Medium industrialized	73
4	Silesian	Highly industrialized (mining)	361
5	Holly Cross	Low industrialized	104

^1)^ Degree of industrialization according to scale developed by Bal-Domańska and Stańczyk [41]

^2)^ Data from 2021 according to Central Statistical Office in Poland (https://stat.gov.pl/)

Hair samples (about 2 g) were cut closes to the skin from the abdomen (from the same place from all animals) within a maximum of 30 min after death of animals (list of samples is. presented in Table S1 in the S1 File). Immediately after collection hair samples were wrapped in aluminum foil and stored in the dark in room temperature until further studies. During collecting and storage samples had no contact with items, that could contain parabens. According to the Act for the Protection of Animals for Scientific or Educational Purposes of 15 January 2015 (Official Gazette 2015, No. 266), applicable in the Republic of Poland the agreement of the ethics committee was not required, because any research activities were not performed on live animals, and samples were collected from wild boars, which are not a protected species legally hunted in the state forests by hunters–members of the Polish Hunting Association, holding appropriate certificates and permissions for hunting.

The number of animals included into the study is limited by the number of legal hunting in Poland, as well as by the willingness of the provincial branches of the Polish Hunting Association union to cooperate in research.

### 2.3. Extraction of parabens from hair and instrumentation

Contaminants from the surface of the hair were removed by the two-fold washing of samples with ultrapure water and two-fold washing in methanol. Then, the hair was dried at 50°C and cut into several-millimeter fragments. The extraction was performed in accordance with the method described by Tzatzarakis et al. and Wojtkiewicz et al. [17, 42]. Briefly, 100 mg of each sample with 2×2 ml of methanol and 25 ng IS in glass screw tubes were extracted in an ultrasonic water bath for 2×2 h with periodic mixing with a vortex system. The extracts were combined and evaporated to dryness under nitrogen steam at 35°C. After adding 100 μL of methanol to the residues, the solution was transferred into 2 mL vials with inserts for liquid chromatography–mass spectrometry (LC-MS) analysis, and 10 μL of the solution was injected into the system.

An LC-MS 2010 EV system (Shimadzu, Kyoto, Japan) was used during analysis. A Supelco Discovery column C18 (250 mm, 4.6 mm, 5 μm; Sigma-Aldrich, St. Louis, MO, USA) was used to analyte separation at a temperature of 30°C. The analysis was performed with a flow rate of 0.6 mL/min using a 5 mM ammonium acetate gradient as solvent A and acetonitrile as solvent B. To monitor the aforementioned substances, we used atmospheric pressure chemical ionization (APCI) and a quadrupole mass filter in negative selected ion monitoring (SIM) mode, with ions m/z 151.05, 194.00 for the MeP, 165.05, 208.10 for the EtP, 179.00, 222.05 for the PrP, 227.10, 270.10 for BeP, 193.05, 236.05 for BuP and 231.05 for the IS. The interface, CDL, and heat block temperatures were set at 400°C, 200°C, and 200°C, respectively; the detector voltage at 1.5 kV; and the nebulizing gas flow at 2.5 L/min.

### 2.4. Method validation and statistical analysis

Analytical parameters to evaluate the efficacy of the methods were tested as follows [43]. Analytical parameters to evaluate the efficacy of the methods were tested as follows. Standard solutions of the analytes were made in the following concentrations: 0, 25, 50, 100, 250, and 500 ng/mL and their linearity was found to be 0.9990 for MeP, 0.9995 for EtP and PrP, 0.9962 for BeP and 0.9918 for BuP. Spiked sample analysis was performed for concentrations of 0, 10, 25, 50, 100, and 250 pg/mg with linearity at 0.9918 for MeP, 0.9989 for EtP, 0.9987 for PrP, 0.9899 for BeP and 0.9933 for BuP.

Both limit of detection (LOD) and limit of quantification (LOQ) were evaluated using the signal to noise ratio. Three repeats of spiked samples (n = 3) were used for the evaluation of the recovery and inter-day precision (%RSD), and four repeats (n = 4) for the evaluation of the accuracy of the method. This was performed using levels 10, 25, 50 and 250 pg/mg for recovery; 10, 25, 50, 100 and 250 pg/mg for accuracy and 25, 100 and 250 pg/mg for precision (Table 2).

**Table 2:** The mean recovery, accuracy, precision (%RSD), linearity and the limits of determination (LOD) and quantification (LOQ) of the applied methodology.

	Mean % recovery	Mean % accuracy	Precision (%RSD)	LOD (pg/mg)	LOQ (pg/mg)	r^2^ (standard curves)	r^2^ (spiked curves)
**MeP**	107.2 ± 10.5	104.6 ± 14.8	20.7	1.8	5.8	0.9990	0.9918
**EtP**	104.6 ± 9.8	100.6 ± 12.1	18.9	2.8	9.2	0.9995	0.9989
**PrP**	94.3 ± 30	109.1 ±10.7	13.8	0.8	2.8	0.9995	0.9987
**BeP**	110.4 ± 35.1	104.3 ±23.5	15.5	0.8	2.7	0.9962	0.9899
**BuP**	118.0 ±18.4	97.2 ±18.6	18.6	0.8	2.6	0.9918	0.9933
**n**	3	4	3	2	2	2	4

MeP–methylparaben, EtP–ethylparaben, PrP–propylparaben, BeP–benzylparaben, BuP -butylparaben, LOD–limit of detection; LOQ–limit of quantification, n- No of repetitions.

As it depicted into the Table 2, the LOQ values were range from 2.6 pg/mg BuP to 9.2 pg/mg for EtP. The mean % recovery of the applied protocol for all tested concentration levels calculated to 94.3% for PrP to 118.0% for BuP, while the corresponding values for the mean accuracy were range from 97.2% for BuP to 109.1 for PrP. The precision values of the method expressed as %RSD were from 13.8 (PrP) to 20.7% (EtP) (Table 2).

GraphPad Prism version 9.2.0 (GraphPad Software, San Diego, California, USA) was used for the statistical analysis. Obtained data were analyzed with descriptive statistics and the following value were calculated: arithmetic mean ± standard deviation (SD), geometric mean ± geometric SD factor, 25% percentile, median 75% percentile values.

Moreover, in evaluations of dependences between paraben levels in the hair of wild boars and animal gender, as well as the human population density and degree of industrialization in the particular voivodeships the nonparametric Mann-Whitney test was used, and the differences were considered as statistically significant at *P*<0.05. Data are presented as mean±standard deviation (SD).

## 3. Results

During the study the presence of at least one paraben was noted in the majority of hair samples included into the investigation (Table 3, Table S1 in the S1 File). Only one sample (sample no. 14) did not contain any of the analyzed parabens (Table S1 in the S1 File). Moreover, the concentration of parabens varied considerably between the particular animals (Table S1 in the S1 File).

**Table 3:** Concentration values (pg/mg) and frequency of detection of parabens (n = 54)–cumulative data.

Compound	Range (pg/mg)	Arithmetic mean±SD (pg/mg)	Geometric mean (pg/mg) ±geometric SD factor	25% percentile (pg/mg)		Median (pg/mg)	75% percentile (pg/mg)	Frequency of detection (%)
MeP	ND-356.4	88.3±72.9	63.3±2.4	36.3	69.3		116.3	96.3
EtP	ND-22.0	17.2±4.8	16.5±1.5	12.4	17.2		22.0	11.1
PrP	ND-26.3	8.5±3.3	8.1±1.4	6.4	7.9		9.7	87.0
BeP	ND-39.9	17.2±12.3	13.0±2.2	7.7	11.4		29.3	44.4
BuP	ND-< LOQ							3.7

MeP–methylparaben, EtP–ethylparaben, PrP–propylparaben, BeP–benzylparaben, BuP -butylparaben, ND–not detected, LOQ—limit of quantification

The most frequently observed paraben was MeP, which presence was confirmed in 96.3% of samples included into the analysis. The presence of MeP was not detected in only two samples included in the study (sample no. 14 and 20) (Table S1 in the S1 File). In one sample (no. 47) the concentration level of this substance was lower than LOQ (5.8 pg/mg) (Table S1 in the S1 File). In other samples MeP concentration levels ranged from 6.3 to 356.4 pg/mg with a mean value 88.28±72.88 pg/mg (Table 3). Another paraben, which presence was found in most of the tested samples was PrP. This substance has been noted in 87% of samples. In samples, where concentration levels of PrP were higher than LOQ (2.8 pg/mg), the levels of this substance fluctuated from 5 to 26.3 pg/mg with a mean value 8.51±3.33 pg/mg (Table 3). In turn the concentration levels of BeP were higher than levels of PrP, and they ranged from 3.6 to 39.9 pg/mg with a mean value 17.15±12.30, but the frequency of detection of this substance was about half less than in the case of PrP Namely, BeP was found in 44.4% of all samples included into the study (Table 3). The frequency of detection of EtP and BuP was clearly lower. The presence of EtP was noted in 11.1% of samples, but its concentration levels higher than LOQ (9.2 pg/mg) were found in only two samples. In turn BuP was observed in only 3.7% of all samples, of which only in one sample the value was higher than LOQ (2.6 pg/mg) (Table 3, Table S1 in the S1 File).

**Fig 2:**
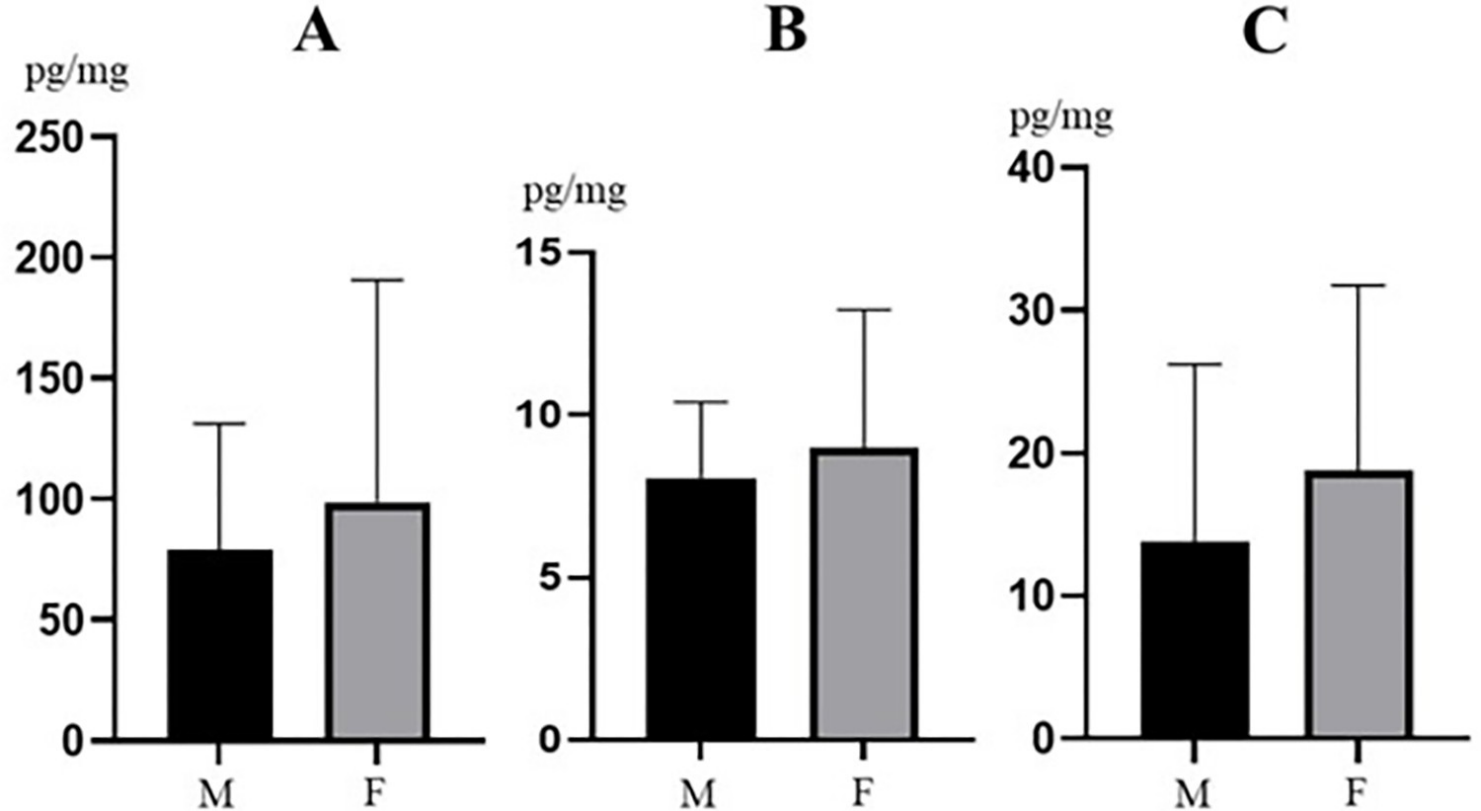
Mean concentration levels (±SD) of methylparaben (**A**), propylparaben (**B**) and benzylparaben (**C**) in wild boar hair samples collected from males (M) and females (F). The figure was created using GraphPad Prism version 9.2.0 (GraphPad Software, San Diego, California USA).

During the present study correlations between the concentration levels of MeP, PrP and BeP and gender of animals, as well as industrialization and human population density were evaluated. The presence of EtP and BuP was found in too few samples to determine such correlations. In males the mean concentration levels (±SD) amounted to 79.00±52.25 pg/mg for MeP, 8.08±2.33 pg/mg for PrP and 13.85±12.39 pg/mg for BeP. In females these values were slightly higher and achieved 98.72±92.06 pg/mg, 9.00±4.26 pg/mg and 18.80±13.00 pg/mg, respectively. However, differences between males and females were not statistically significant (Fig 2).

**Fig 3:**
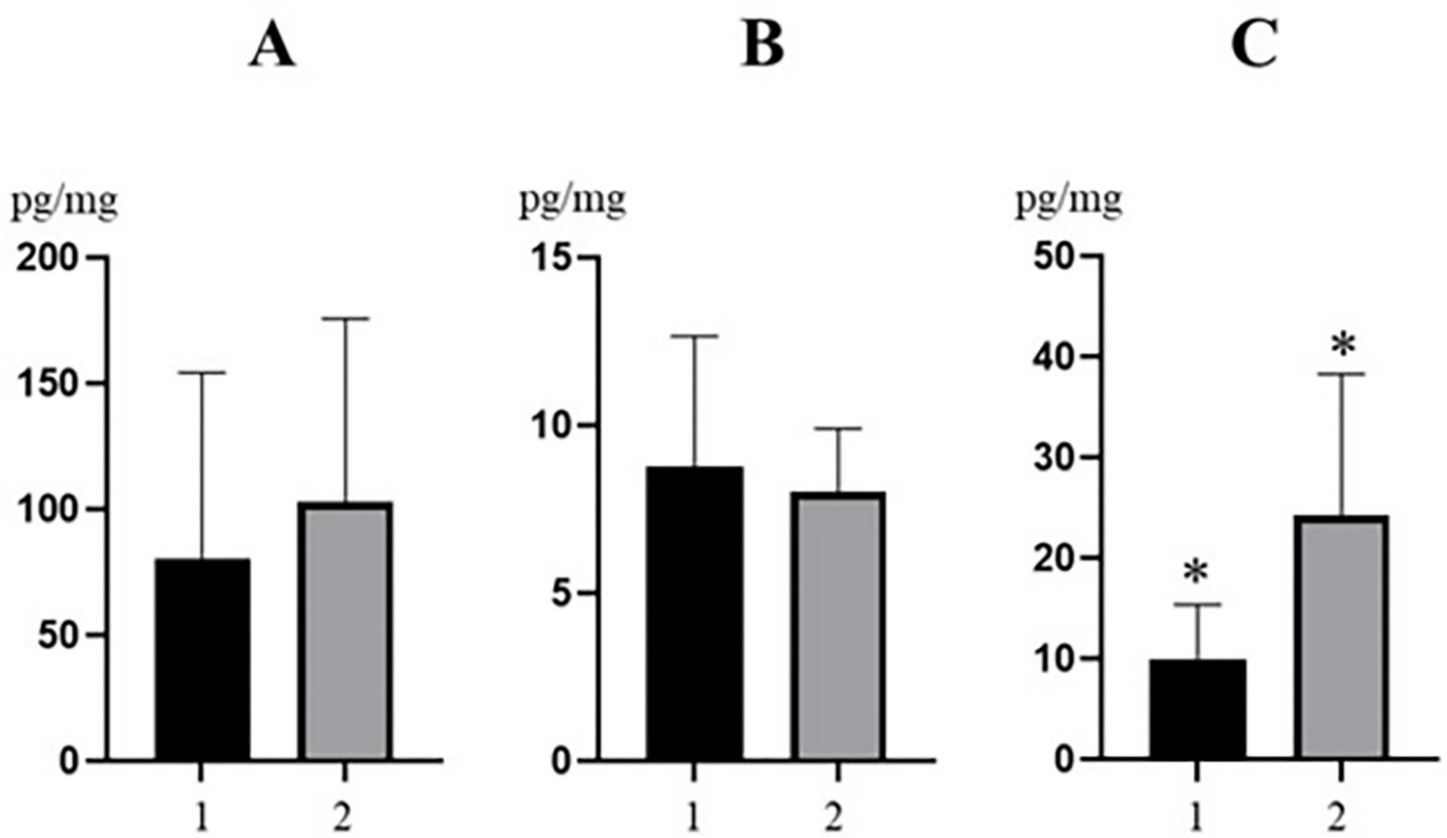
Mean concentration levels (±SD) of methylparaben (**A**), propylparaben (**B**) and benzylparaben (**C**) in wild boar hair samples collected in voivodeships with a low and medium degree of industrialization and lower human population density (1) and in voivodeships with a high degree of industrialization and higher human population density (2). Statistically significant differences (P≤0.05) are marked with *. The figure was created using GraphPad Prism version 9.2.0 (GraphPad Software, San Diego, California USA).

In voivodeships with a high and very high degree of industrialization and a higher human population density (Silesian and Pomeranian), the mean concentration levels amounted to 102.9±72.83 pg/mg for MeP, 8.02±1.89 pg/mg for Prp and 24.28±14.05 pg/mg for BeP. In voivodeships with medium and low degree of industrialization and a lower human population density (Kuyavian-Pomeranian, West Pomeranian and Holy Cross) these values amounted to 80.28±73.89 pg/mg, 8.76±3.88 pg/mg and 10.02±5.37 pg/mg, respectively (Fig 3). Only in the case of BeP differences in concentration levels between higher industrialized areas with higher human population and areas with lower industrialization and lower human population density were statistically significant (Fig 3).

## 4. Discussion

The results obtained during the present study have shown that parabens are present in hair samples collected from wild boars living in all regions studied. This proves not only that wild terrestrial mammals are exposed to these substances, but also the usefulness of hair for biomonitoring of parabens in wild animals. It should be underlined that until now the use of hair as a matrix for research on the degree of exposure to parabens has been limited only in humas and dogs (Table 4).

**Table 4:** Concentration levels of parabens in hair samples (pg/mg) noted in previous studies.

Species	Country	n	MeP	EtP	PrP	BeP	BuP	References
Humans	Belgium	114	<LOQ-53356.0	<LOQ-26967.7	<LOQ-1517.1			[36]
	France	5	0.365–318.536	<LOQ-1.407	<LOQ-291.744		<LOQ-0.874	[34]
	Germany	4		810–1980	400–1520			[44]
	Greece	95	17.6–27437.0	11.0–4224.5		2.1–66.6	1.8–2513.7	[45]
	Korea	10	48.3–224.2	11.5–158.3	70.2–214.5	15.3–100.2	25.4–111.1	[32]
	Poland	30	87.2–42430.1	19.1–8413.1	18.9–11150.3	36.0–700.7	3.8–2636.0	[17]
	Spain	6	78–624	7.0–42	27–238			[46]
		6	10.2–33	9.0	11.6–107		3.5–9.4	[47]
		42	68.3–14187	2.9–6565	12.5–9009			[33]
Dogs	Poland	30	<LOQ-1023	<LOQ-382	8.12–527			[22]

MeP–methylparaben, EtP–ethylparaben, PrP–propylparaben, BeP–benzylparaben, BuP -butylparaben, LOQ—limit of quantification, *n*—number of samples included into the study

Despite a relatively small number of studies on the content of parabens in the hair, it is known that such samples are a good alternative to classic matrices, such as blood serum and urine [33, 36]. Hair samples are of particular importance in the study on long-term, environmental exposure to parabens. In the urine or serum levels of these substances are subject to frequent fluctuation with the increase in the concentration levels after exposure and their decrease after a relatively short period of time [48]. Previous studies have shown that even after a single exposure, the levels of parabens may increase in the serum and urine, and after some hours or days, due to the fact that parabens are substances with short elimination half-lives, these levels may decrease significantly [34]. Such pronounced fluctuations make long-term exposure to parabens much more difficult to establish and require repeated sampling over time [48, 49]. Contrary to urine or blood serum parabens accumulate in the hair, and their concentration levels in this matrix well reflect long-term exposure. Moreover, hair samples are they are easy to collect, store and transport.

Of course, the hair as a matrix to study on exposure to parabens is not without flaws. The main one is that the concentration levels of substances contaminated the hair consist of two components [50]. The first of them are substances which get into the interior of the body, reach the hair roots through capillaries and penetrate from the blood [50, 51]. The second part of substances found in the hair penetrate inside the hair directly from external environment [51, 52]. Unfortunately during hair samples analysis it is impossible to determine, whether the substance comes from internal or external exposure [51]. It should be underlined that external exposure directly from the environment is more important in humans, because human hair often comes into direct contact with cosmetics and personal care products, as well as other objects, which may contain parabens [53]. In the case of wild animals penetration of parabens from air into hair seems to be of marginal importance. Although on the other hand, wild boars often live near human settlements and feed and sleep in landfills [54, 55] and therefore in cannot be excluded that in some individuals external exposure may influence on the concentration levels of parabens in the hair. The next limitation of hair samples analysis is that, due to the accumulation of parabens in the hair for long time, the hair samples are useless for studying short-term changes in exposure to these substances [49].

It should be underlined that till now hair samples analysis has not been used to study on parabens concentration levels in wild animals. Moreover, the majority of previous studies on this issue concern marine animals and used various tissues as a matrix, in which parabens have been evaluated, including blood plasma, liver, kidney, brain, muscles and others (Table S2 in the S1 File). Previous studies have also shown that parabens concentration levels significantly depend on the species animal, part of the world, where investigations have been made, kind of matrix and type of paraben studied (Table S2 in the S1 File).

The comparison of results obtained in present study with previous investigations on wild animals is difficult, due to the fact that previous observations have been made on other animal species, other matrices and in other parts of the world. Moreover, the majority of previous studies have been performed on very small groups, the size of which often does not exceed a few individuals (Table S2 in the S1 File). Nevertheless, despite the above-mentioned differences between the current study and previous studies on wild animals, it can be concluded that the exposure to parabens is significant. This is especially visible in the case of parabens other than MEP, which in most previous studies, they were not present in the samples from wild animals or were found in trace amounts (Table S2 in the S1 File). Analyzing previous publications on wild animals (Table S2 in the S1 File), the above-mentioned differences in paraben levels depending on the place of research and animal species studied are very clearly visible. In some regions and some species (especially water species) the maximum paraben concentration levels are several to even several hundred times higher than those noted in this study. On the other hand, in other studies paraben concentration levels were significantly lower than the values ​​observed in this study (Table S2 in the S1 File).

The only previous work on parabens in wild animals in Poland concerns studies on wild bat guano [29], in which MeP, EtP, PrP and BuP have been found (Table S2 in the S1 File). This observation together with results obtained in present investigation and studies on parabens in the elements of the natural environment [56, 57] have indicated that environment in Poland is polluted with parabens to a relatively considerable extent, what may translate to the risk of exposure to these substances not only in humans [17, 58] and domestic animals [22], but also in wild animals [29]. Moreover, the presence of parabens in the wild boar hair may be connected with the lifestyle of this animal species. More and more often wild boars live in close proximity of humans sites (to the extent that they can be found in city centers) and forage in the landfills dumpsters, which undoubtedly has a huge impact on the degree of exposure to anthropogenic pollutants [59, 60].

However, in spite of such lifestyle the concentration levels of parabens in the wild boar hair samples are clearly lower than those noted in the majority studies on hair in humans (Table 4). It is connected with the fact that humans are exposed to parabens, which are anthropogenic pollutants, to a much greater extent than animals through the everyday use of cosmetics, personal care products, medicines and food containers [2, 8].

The present study has shown that among parabens MeP–a paraben with the shortest functional group is a substance, to which wild animals are most exposed. It is in agreement with previous observations, which have described that MeP is the paraben most commonly and abundantly present in the elements of the natural environment [56, 57], humans (Table 4), as well as wild (Table S2 in the S1 File) and domestic animals [22]. However, due to the relatively small number of studies on environmental pollution with parabens in Poland, it is difficult to determine what is the main source of exposure of wild boars to these substances. It can only be supposed that the main source of exposure of wild boars to parabens is water and food, although (as mentioned above) contact exposure to the skin cannot be ruled out.

During this investigation dependences between paraben levels in the hair of wild boars and animal gender, as well as the human population density and degree of industrialization were also studied. Such research is justified, because previous studies on the levels of various endocrine disrupting chemicals in biological samples have shown intragender differences [61, 62]. However, the results on this issue are often contradictory and the reason for such differences is not clear [61, 63, 64]. In turn, differences in paraben levels in biological samples depending on the place, where studies have been conducted are relatively well known (Table 4, Table S2 in the S1 File). These differences may be connected with the degree of industrialization and urbanization, and the aim of the present analysis was to investigate, whether urbanization and industrialization may affect the degree of wild boar exposure on parabens.

In this study MeP, PrP and BeP concentration levels were a slight higher in females, but there were no statistically significant intragender differences in these values and values noted in males. Such differences are relatively well documented in humans, where higher concentration levels of parabens have been noted in women [33, 58, 65, 66]. However, it seems that these differences noted in humans may result from the fact that women relatively more often than men use products containing parabens including cosmetics and personal care products. In turn, a slight higher concentration levels of parabens in females noted in the present study may result from the fact that wild boar females are less timid and more often stay in close proximity to human settlements than males. Of course various parabens concentration levels in males and females may also result from intragender differences in hormonal activity, general metabolism rate and activity of factors taking part in parabens metabolism. This is all the more likely that intragender differences in parabens have been noted in domestic animals [22], as well as various effects of parabens in particular genders have been observed [67, 68].

Although previous investigations have reported correlations between industrialization and urbanization and paraben levels in the environment [8, 23, 69], the results of present study are not clear. Namely, such statistically significant correlations have been found only in the case of BeP. Probably this is due to the fact that the other parabens included into the study (MeP and PrP) are more often used in industry and everyday objects, and therefore they more often pollute the environment also in less urbanized areas, what have been confirmed in previous works [56].

The question arises, whether the doses of parabens to which wild boars are exposed and causing observed concentration levels in the hair samples may negatively affect the health status of animals. The answer is rather difficult. On the one hand negative health effects of parabens are still discussed, although more and more studies present toxic activity of these substances [2, 8]. Moreover, the toxic mechanisms of parabens are not well understood. It cannot be ruled out that these mechanisms, as well as metabolism of parabens are various in various animal species as is the case in other synthetic organic endocrine disruptors [70, 71]. On the other side some studies have reported negative effects of parabens even in relatively small doses, especially if they act on the living organisms together with other pollutants commonly occurring in the natural environment, including among others bisphenol A or pesticides [72]. This is because of synergistic and/or additive interactions between various chemicals [73]. Given this, it cannot be excluded that parabens may negatively affect the wild animals.

## 5. Conclusion

During the present study the hair samples have been used to biomonitoring of parabens in wild boars. The obtained results indicated that this animal species are exposed to parabens, especially to MeP, PrP and BeP which have been detected in 96.3%, 87.0% and 44.5% of samples included into the study, respectively. In turn EtP and BuP seem to be less important, when it comes to the exposure of wild boars to parabens. Due to the relatively high exposure of wild boars to parabens, it can be assumed that these compounds may affect the health status of these animals. However, the exact explanation of this issue require further studies on toxicity and metabolism of these substances in wild boars. Moreover, current research also confirms the usefulness of hair samples for studies on the degree of exposure of wild animals to parabens, especially in studies on long-term exposure to these compounds.

## Supporting information

S1 File (DOCX)
