# Supplementary material for: Biomonitoring of parabens in wild boars through hair samples analysis
Source: PLoS One. 2024 Feb 21;19(2):e0297938. doi: 10.1371/journal.pone.0297938 (PMC10880979; doi:10.1371/journal.pone.0297938)
Supplement: S1 File — (DOCX) [file pone.0297938.s001.docx]

**Supplementary materials to :**

**Biomonitoring of parabens in wild boars through hair samples analysis**

**Sławomir Gonkowski^1^, Manolis Tzatzarakis^2^, Elena Vakonaki ^2^, Elena Meschini ^2^,** **László Könyves^3^, Liliana Rytel^3^**

^1^ Department of Clinical Physiology, Faculty of Veterinary Medicine, University of Warmia and Mazury in Olsztyn, Oczapowskiego 13, 10-957 Olsztyn, Poland.

^2^ Laboratory of Toxicology, School of Medicine, University of Crete, 71003 Heraklion, Crete, Greece.

^3^ Department of Animal Hygiene, Herd Health and Mobile Clinic, University of Veterinary Medicine, 1078 Budapest, Hungary

^4^ Department and Clinic of Internal Diseases, Faculty of Veterinary Medicine, University of Warmia and Mazury in Olsztyn, Oczapowski Str. 14, 10-718 Olsztyn, Poland

Table S1 Wild boar hair samples characterization and parabens concentration levels (pg/mg dry weight) in particular samples

| No | Animal gender | Voivodeship | Hair length (cm) | MeP | EtP | PrP | BeP | BuP |
| --- | --- | --- | --- | --- | --- | --- | --- | --- |
| 1 | Male | Kuyavian-Pomeranian | 6 | 69.3 | ND | 6.5 | <LOQ | ND |
| 2 | Male | Kuyavian-Pomeranian | 6 | 106.8 | ND | 7.0 | ND | ND |
| 3 | Female | Kuyavian-Pomeranian | 6 | 215.8 | ND | 8.0 | ND | ND |
| 4 | Male | Kuyavian-Pomeranian | 7 | 96.1 | ND | 8.8 | ND | ND |
| 5 | Male | Kuyavian-Pomeranian | 7 | 80.8 | ND | 5.9 | 10.2 | ND |
| 6 | Female | Kuyavian-Pomeranian | 7 | 151.7 | ND | 5.8 | ND | ND |
| 7 | Female | Kuyavian-Pomeranian | 6 | 244.1 | ND | 26.3 | 8.1 | ND |
| 8 | Male | Kuyavian-Pomeranian | 8 | 85.4 | ND | 10.5 | 8.7 | ND |
| 9 | Male | Kuyavian-Pomeranian | 10 | 128.9 | ND | 6.0 | ND | ND |
| 10 | Female | Kuyavian-Pomeranian | 9 | 356.4 | ND | 11.1 | 16.8 | 9.5 |
| 11 | Male | Silesian | 5 | 116.3 | ND | 11.5 | ND | ND |
| 12 | Male | Silesian | 5 | 44.8 | ND | 11.2 | 38.4 | ND |
| 13 | Male | Silesian | 6 | 79.8 | ND | 6.9 | 12.7 | ND |
| 14 | Male | Silesian | 6 | ND | ND | ND | ND | ND |
| 15 | Female | Silesian | 6 | 117.4 | ND | 9.4 | 39.9 | ND |
| 16 | Female | Silesian | 5 | 107.6 | ND | 8.8 | ND | ND |
| 17 | Male | Silesian | 7 | 43.3 | 12.4 | 7.3 | ND | ND |
| 18 | Male | Silesian | 4 | 155.8 | ND | 5.6 | ND | ND |
| 19 | Female | Silesian | 12 | 287.8 | ND | 6.4 | ND | ND |
| 20 | Female | Silesian | 7 | ND | ND | 5.9 | 6.4 | ND |
| 21 | Female | Pomeranian | 8 | 15.5 | ND | 6.9 | ND | ND |
| 22 | Male | Pomeranian | 6 | 244.3 | 22.0 | ND | ND | ND |
| 23 | Female | Pomeranian | 3 | 110.9 | ND | ND | 33.6 | ND |
| 24 | Female | Pomeranian | 2 | 86.1 | ND | ND | 27.9 | ND |
| 25 | Male | Pomeranian | 4 | 121.0 | ND | 9.7 | ND | ND |
| 26 | Female | Pomeranian | 3 | 22.9 | ND | 8.4 | 17.5 | ND |
| 27 | Female | Pomeranian | 7 | 14.5 | ND | 7.9 | 5.0 | ND |
| 28 | Male | Pomeranian | 8 | 114.0 | <LOQ | ND | ND | ND |
| 29 | Female | Pomeranian | 8 | 53.4 | ND | ND | ND | ND |
| 30 | Female | Pomeranian | 5 | 117.4 | ND | 6.4 | 37.1 | ND |
| 31 | Female | West Pomeranian | 6 | 63.8 | ND | 8.4 | 20.2 | ND |
| 32 | Female | West Pomeranian | 6 | 34.3 | <LOQ | 8.3 | ND | ND |
| 33 | Male | West Pomeranian | 6 | 31.1 | ND | 5.1 | ND | ND |
| 34 | Male | West Pomeranian | 4 | 36.3 | ND | 6.8 | ND | ND |
| 35 | Male | West Pomeranian | 4 | 112.7 | <LOQ | 5.0 | ND | ND |
| 36 | Male | West Pomeranian | 8 | 50.0 | ND | 6.0 | ND | ND |
| 37 | Female | West Pomeranian | 9 | 59.9 | ND | 5.7 | ND | ND |
| 38 | Male | West Pomeranian | 9 | 43.7 | ND | 7.3 | ND | ND |
| 39 | Male | West Pomeranian | 9 | 126.3 | ND | 6.1 | 3.6 | ND |
| 40 | Female | West Pomeranian | 9 | 97.7 | <LOQ | 6.2 | 8.8 | ND |
| 41 | Female | Holy Cross | 9 | 31.1 | ND | 7.9 | ND | ND |
| 42 | Female | Holy Cross | 4 | 14.5 | ND | 7.6 | <LOQ | ND |
| 43 | Male | Holy Cross | 4 | 29.5 | ND | 8.2 | ND | ND |
| 44 | Male | Holy Cross | 8 | 27.7 | ND | 6.9 | <LOQ | ND |
| 45 | Male | Holy Cross | 12 | 40.9 | ND | 10.7 | <LOQ | ND |
| 46 | Male | Holy Cross | 5 | 6.3 | ND | 11.4 | ND | ND |
| 47 | Male | Holy Cross | 7 | <LOQ | ND | ND | ND | ND |
| 48 | Female | Holy Cross | 5 | 21.1 | ND | 10.4 | <LOQ | ND |
| 49 | Female | Holy Cross | 3 | 56.0 | ND | 9.4 | ND | ND |
| 50 | Male | Holy Cross | 3 | 47.7 | ND | 13.4 | ND | ND |
| 51 | Female | Holy Cross | 6 | 44.2 | ND | 11.0 | ND | ND |
| 52 | Male | Holy Cross | 8 | 84.6 | ND | 9.6 | <LOQ | ND |
| 53 | Female | Holy Cross | 8 | 45.1 | ND | 11.8 | 4.3 | ND |
| 54 | Male | Holy Cross | 7 | 9.5 | ND | 8.6 | 9.5 | <LOQ |

MeP – methylparaben, EtP – ethylparaben, PrP – propylparaben, BeP – benzylparaben, BuP -butylparaben,, ND – not detected; <LOQ: below the limit of quantification of the method

Table S2 Selected previous studies on paraben concentration levels in wild animals. Paraben concentration levels are shown in ng/g (in solid matrices) or ng/mL (in liquid matrices).

| Country/  Localisation | Species | matrix | n | Concentration levels | | | |  | Ref. |
| --- | --- | --- | --- | --- | --- | --- | --- | --- | --- |
|  |  |  |  | MeP | EtP | PrP | BuP | BeP |  |
| Antarctica | Clams | body | 7 | <2.1-5.8 | n.d. | n.d.-5.3 | n.d. |  | [1] |
|  | Sea urchin | body | 1 | 5.7 | n.d. | n.d. | n.d. |  |  |
|  | Fish | body | 7 | 5.1-26.9 | n.d. | n.d | n.d. |  |  |
|  |  | liver | 1 | 2.4 | n.d. | n.d. | n.d. |  |  |
| China /Yangtze river | Fish | plasma | 36 | 11.6–39.5 | 0–10.5 | 0–6.97 | 0–<1.46 |  | [2] |
|  |  | bile | 35 | 8.17–21.9 | 0–31.6 | 2.19–112 | 0–4.42 |  | [3] |
| China /Taihu Lake |  | muscle | 199 | 88.1-1200 | 33.6-450 | 55.3-543 | <LOQ-40.0 | <LOQ-264 | [4] |
| Colombia | Fish | muscle | 20 | <20-32.0 |  |  |  |  | [5] |
| Korea/coastal waters | Dolphin | muscle | 12 | 12-121 |  |  |  |  | [6] |
|  |  | kidney | 6 | 181-359 |  |  |  |  |  |
|  |  | stomach | 12 | 44-228 |  |  |  |  |  |
|  |  | liver | 12 | 13-569 |  |  |  |  |  |
|  |  | brain | 5 | 4.2-50 |  |  |  |  |  |
|  |  | uterus | 2 | 37-74 |  |  |  |  |  |
| Poland | Bat | guano | 40 | 14.00–142.00 | <0.05–239 | <0.05–229 | <0.02–27.5 |  | [7] |
| Spain | Fish | muscle | 59 | n.d.-84.69 | n.d.-0.82 | n.d.-7.43 |  |  | [8] |
| USA/Gulf of Mexico | dolphin | liver | 17 | <41.1-865 | n.d. | <2.05-3.47 | n.d. | n.d. | [9] |
| USA/Atlantic ocean | whale | liver | 3 | <20.5-37.7 | n.d. | <4.10 | n.d. | n.d. |  |
| USA/west coast | Sea otter | liver | 53 | n.a.-686 | n.d.-31.6 | n.d.-3.95 | n.d.-31.8 | n.d. |  |
|  |  | kidney | 10 | n.a.-360 | n.d. | n.d. | n.d. | n.d. |  |
|  |  | brain | 10 | 5.99-77.2 | <1.03-3.26 | n.d. | n.d. | n.d. |  |
| USA /Alaska | Polar bear | liver | 10 | <4.10-16.9 | n.d. | n.d. | n.d. | n.d. |  |
| USA /Greater Pittsburgh Area | Fish | brain | 58 | n.d. | n.d. | n.d. | n.d. |  | [10] |
| USA/Florida | Fish | liver | 6 | <2.01-44.3 | n.d. | n.d. | n.d. | n.d. | [11] |
|  |  | muscle | 6 | n.a-43.9 | n.d. | n.d. | n.d. | n.d. |  |
|  |  | kidney | 1 | 18.8 | n.d. | n.d. | n.d. | n.d |  |
|  |  | gill | 1 | 71 | n.d. | n.d. | n.d. | n.d. |  |
|  |  | brain | 1 | 735 | n.d. | n.d. | n.d. | n.d. |  |
| USA/Michigan | Black bear | liver | 2 | 33.5 - 58.2 | n.d. | n.d. | n.d. | n.d. |  |
|  |  | kidney | 2 | 24.0 - 37.6 | n.d. | n.d. | n.d. | n.d. |  |
|  | Bald eagle | liver | 1 | 796 | n.d. | n.d. | n.d. | n.d. |  |
|  |  | plasma | 15 | <0.2–0.37 | n.d. | n.d. | n.d. | n.d. |  |
|  |  | kidney | 1 | 580 | n.d. | n.d. | n.d. | n.d. |  |
|  |  | muscle | 2 | 87.4- 169 | n.d. | n.d. | n.d. | n.d |  |
|  | Herring gull | eggs | 9 | <1.99-14.0 | n.d. | n.d. | n.d. | n.d. |  |

MeP – methylparaben, EtP – ethylparaben, PrP – propylparaben, BuP -butylparaben, BeP – benzylparaben, LOQ - limit of quantification, *n* - number of samples included into the study, n.d. – not detected.

References:

1. Emnet P, Gaw S, Northcott G, Storey B, Graham L. Personal care products and steroid hormones in the Antarctic coastal environment associated with two Antarctic research stations, McMurdo Station and Scott Base. Environ. Res. 2015; 136: 331-42.
2. Yao L, Lv YZ, Zhang LJ, Liu WR, Zhao JL, Yang YY, et al. Bioaccumulation and risks of 24 personal care products in plasma of wild fish from the Yangtze River, China. Sci. Total Environ. 2019; 665: 810–819.
3. Yao L, Lv YZ, Zhang LJ, Liu WR, Zhao JL, Liu YS, et al. Determination of 24 personal care products in fish bile using hybrid solvent precipitation and dispersive solid phase extraction cleanup with ultrahigh performance liquid chromatography-tandem mass spectrometry and gas chromatography-mass spectrometry. J. Chromatogr. A. 2018; 1551: 29–40.
4. Wang N, Hu X, Lu S, Ma S, Kang L, Liao S, et al. Interrelationship of anthropogenic activity and parabens in fish from Taihu Lake during 2009–2017. Environ. Pollut. 2019; 252 Pt B: 1002–1009.
5. Cacua-Ortiz SM, Aguirre NJ, Peñuela GA. 2020. Methyl paraben and carbamazepine in water and striped catfish (Pseudoplatystoma magdaleniatum) in the Cauca and Magdalena Rivers. Bull. Environ. Contam. Toxicol. 2020; 105: 819–826.
6. Jeong Y, Xue J, Park KJ, Kannan K, Moon HB. Tissue-specific accumulation and body burden of parabens and their metabolites in small cetaceans. Environ. Sci. Technol. 2019;53:475–481.
7. Gonkowski S, Martín J, Aparicio I, Santos JL, Alonso E, Rytel L. 2023. Evaluation of parabens and bisphenol A concentration levels in wild bat guano samples. Int. J. Environ. Res. Public Health. 2023; 20: 1928.
8. Pico Y, Belenguer V, Corcellas C, Diaz-Cruz MS, Eljarrat E, et al. Contaminants of emerging concern in freshwater fish from four Spanish Rivers. Sci. Total. Environ. 2019; 659: 1186–1198.
9. Xue J, Sasaki N, Elangovan M, Diamond G, Kannan K. Elevated accumulation of parabens and their metabolites in marine mammals from the United States coastal waters. Environ. Sci. Technol. 2015; 49: 12071–12079.
10. Renz L, Volz C, Michanowicz D, Ferrar K, Christian C, Lenzner D, et al. A study of parabens and bisphenol A in surface water and fish brain tissue from the Greater Pittsburgh Area. Ecotoxicology. 2013; 22: 632–641.
11. Xue J, Kannan K. Accumulation profiles of parabens and their metabolites in fish, black bear, and birds, including bald eagles and albatrosses. Environ. Int. 2016; 94: 546–553.
